# The Greig cephalopolysyndactyly syndrome

**DOI:** 10.1186/1750-1172-3-10

**Published:** 2008-04-24

**Authors:** Leslie G Biesecker

**Affiliations:** 1 Genetic Disease Research Branch, National Human Genome Research Institute, National Institutes of Health, Bethesda, MD, USA

## Abstract

The Greig cephalopolysyndactyly syndrome (GCPS) is a pleiotropic, multiple congenital anomaly syndrome. It is rare, but precise estimates of incidence are difficult to determine, as ascertainment is erratic (estimated range 1–9/1,000,000). The primary findings include hypertelorism, macrocephaly with frontal bossing, and polysyndactyly. The polydactyly is most commonly preaxial of the feet and postaxial in the hands, with variable cutaneous syndactyly, but the limb findings vary significantly. Other low frequency findings include central nervous system (CNS) anomalies, hernias, and cognitive impairment.

GCPS is caused by loss of function mutations in the *GLI3 *transcription factor gene and is inherited in an autosomal dominant pattern. The disorder is allelic to the Pallister-Hall syndrome and one form of the acrocallosal syndrome.

Clinical diagnosis is challenging because the findings of GCPS are relatively non-specific, and no specific and sensitive clinical have been delineated. For this reason, we have proposed a combined clinical-molecular definition for the syndrome. A *presumptive *diagnosis of GCPS can be made if the patient has the classic triad of preaxial polydactyly with cutaneous syndactyly of at least one limb, hypertelorism, and macrocephaly. Patients with a phenotype consistent with GCPS (but which may not manifest all three attributes listed above) and a *GLI3 *mutation may be diagnosed definitively with GCPS. In addition, persons with a GCPS-consistent phenotype who are related to a definitively diagnosed family member in a pattern consistent with autosomal dominant inheritance may be diagnosed definitively as well. Antenatal molecular diagnosis is technically straightforward to perform.

Differential diagnoses include preaxial polydactyly type 4, the GCPS contiguous gene syndrome, acrocallosal syndrome, Gorlin syndrome, Carpenter syndrome, and Teebi syndrome.

Treatment of the disorder is symptomatic, with plastic or orthopedic surgery indicated for significant limb malformations.

The prognosis for typically affected patients is excellent. There may be a slight increase in the incidence of developmental delay or cognitive impairment. Patients with large deletions that include *GLI3 *may have a worse prognosis.

The Article is a work of the United States Government. Title 17 U.S.C 5 105 provides that copyright protection is not available for any work of the United States Government in the United States. The United States hereby grants to anyone a paid-up, nonexclusive, irrevocable worldwide license to reproduce, prepare derivative works, distribute copies to the public and perform publicly and display publicly the work, and also retains the nonexclusive right to do all of the above for or on behalf of the United States.

## Disease name, synonyms, and included diseases

Greig cephalopolysyndactyly (GCPS) syndrome is named after David Middleton Greig for his 1926 manuscript describing a patient with this disorder [1]. Although the name is commonly confused with that of Grieg, the Norwegian composer, Greig was a Scot, whose name is pronounced Gregg (with a trilled "r"). The term "Greig syndrome" is not favored as it denotes a less specific dyad of hypertelorism and macrocephaly [2]. The alternative term "polysyndactyly with peculiar skull shape" is disparaging and should not be used. Hootnick-Holmes syndrome has been suggested to be the same entity [2]. Note that the clinical diagnostic entity of GCPS is clearly distinct from Pallister-Hall syndrome, which is allelic. GCPS should also be distinguished from acrocallosal syndrome, although this distinction can be difficult or impossible without the utilization of molecular diagnostics.

## Definition

The Greig cephalopolysyndactyly syndrome (GCPS) is a rare, pleiotropic, multiple congenital anomaly syndrome characterized by the primary clinical triad of polysyndactyly, macrocephaly, and hypertelorism.

## Epidemiology

The incidence of GCPS is difficult to estimate. It is impossible to determine the incidence of a disorder for which there are no reliable clinical criteria and molecular diagnostics are not yet in wide use. As the disorder blends in a phenotypic continuum with non-syndromic polydactyly, it may be much more prevalent than it seems. This author estimates that it is in the 1–9/1,000,000 range.

## Clinical description

**Figure 1:**
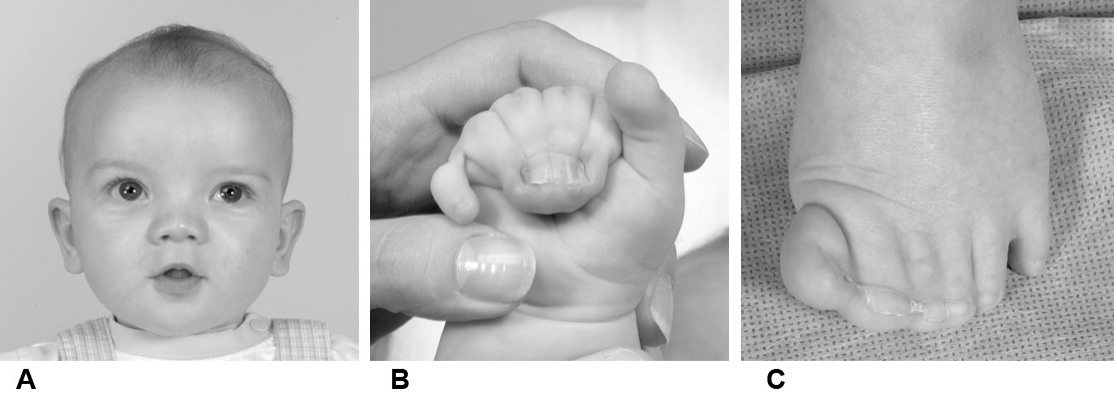
**A patient with Greig cephalopolysyndactyly [26].** A. Facial view of the patient. Note the hypertelorism and macrocephaly. B. The hand of this patient shows a broad thumb, complete cutaneous syndactyly of digits 2–5 with fusion of the nails and a postaxial supernumerary digit. C. The foot of this patient shows a partially duplicated hallux with cutaneous syndactyly of several digits.

The primary clinical triad of GCPS is polysyndactyly, macrocephaly, and hypertelorism [2-4] (Figure 1). The polydactyly is classically described as preaxial, and may occur in any limb. Postaxial polydactyly may be more common than preaxial and in our experience, the most common finding is postaxial polydactyly of the hands and preaxial polydactyly of the feet. The severity of the polydactyly varies widely, among individuals and among limbs in the same individual. This can vary from an apparently normal extremity, through subtle broadening of the thumb or hallux, tiny postaxial nubbins, to partially bifid digits, hypoplastic supernumerary digits, fully formed supernumerary digits, and higher order polydactyly (this author has seen a single patient with GCPS and octadactyly). The cutaneous syndactyly is also highly variable. Many patients have none. Some patients have mild partial cutaneous syndactyly of a few digits and this spectrum continues through to complete cutaneous syndactyly of all digits, not unlike that seen in patients with Apert syndrome.

The craniofacial manifestations are also highly variable. Some patients have significant hypertelorism (increased interpupillary distance) with or without telecanthus (increased inner canthal distance). Some patients also have macrocephaly, which is not typically associated with central nervous system (CNS) anomalies (see section below on GCPS-contiguous gene syndrome for exceptions to this). The craniofacial findings of GCPS commonly cause challenges in clinical diagnosis. First, mild hypertelorism is generally considered an attractive trait, and it may be missed by examiners not trained to recognize it. Second, because familial macrocephaly is common, this too may be missed by inexperienced examiners. Finally, all of these craniofacial findings are highly variable and we have evaluated a number of multiplex GCPS families where some affecteds had clear craniofacial findings and some had nearly none.

Other, less common anomalies in GCPS include craniosynostosis, mental retardation, agenesis of the corpus callosum, and umbilical and diaphragmatic hernias. In our experience, mental retardation is not common patients with typical GCPS. Of course, the background rate of cognitive impairment in the population is 2–3%, so it is difficult to prove that a rare malformation syndrome has an elevated or baseline risk for this complication, if only a few hundred cases have been analyzed. We counsel families that it is not clear at this time that GCPS has a significantly higher rate of cognitive impairment than the background risk. Instead, most of the risk of cognitive impairment seems to be associated with the GCPS-contiguous gene syndrome (see below). Craniosynostosis was noted in several of the earliest GCPS case reports, but we have observed this in very few patients.

There have been several case reports of patients with GCPS and tumors, such as leukemia and gliomas [5]. Again, it is difficult to assign relative risks for such a putative association, especially considering ascertainment biases.

More generally, ascertainment bias likely plays a major role in distorting the reported frequencies of many manifestations of GCPS. Because patients enrolled in most clinical and molecular research studies must fulfill certain eligibility criteria, this biases case series toward more severely affected patients. This author suspects that there are many individuals with mild GCPS who instead carry a diagnosis of non-syndromic polydactyly, because they were not evaluated by a clinician familiar with the subtleties of this disorder.

## Etiology

More than 75% of patients with clinically recognizable GCPS who have been evaluated in the NIH study have been found to have mutations in *GLI3 *[6]. GCPS is caused by mutations that lead to haploinsufficiency for *GLI3 *and, as is typical for this mode of pathogenesis, the spectrum of mutations is very large. We suspect that there are patients among those who are *GLI3 *mutation-negative who have cryptic mutations in this gene. However, the gene is large (about 300 kb) and screening for all possible null mutations is nearly impossible. It remains a possibility that some patients with a GCPS phenotype could have mutations in genes other than *GLI3*, and we hypothesize that mutations in other genes in the GLI-Sonic Hedgehog family could cause such a phenotype.

Mutations shown to cause GCPS include nonsense, missense, and splicing mutations, and translocations, deletions and insertions [6-15]. The deletions range from a single nucleotide to nearly a megabase in size. The range of insertions is not so broad, ranging from a single nucleotide to ~60 kb. A useful rule is that mutations that are smaller than about 1 Mb generally cause typical GCPS whereas mutations larger than this cause what we have termed the GCPS-contiguous gene syndrome (GCPS-CGS). This latter disorder is best considered as a distinct clinical diagnosis. In our experience, such patients have a high frequency of cognitive impairment and seizures. In addition, many of these patients have a hypoplastic corpus callosum and a normal or small head circumference. We have suggested that these manifestations are non-specific and caused by the loss of numerous contiguous genes [9].

## Diagnostic criteria

It is difficult to precisely define GCPS on clinical grounds because the major manifestations overlap substantially with other disorders (see differential diagnosis, below). For this reason, we have proposed a combined clinical-molecular definition. A *presumptive *diagnosis of GCPS can be made if the patient has the classic triad of preaxial polydactyly with cutaneous syndactyly of at least one limb, hypertelorism, and macrocephaly. The presumptive criteria could be useful to identify patients who may benefit from undergoing molecular analysis of *GLI3*. Patients with a phenotype consistent with GCPS and a *GLI3 *mutation may be diagnosed definitively with GCPS. In addition, persons with a GCPS-consistent phenotype who are related to a definitively diagnosed family member in a pattern consistent with autosomal dominant inheritance may be diagnosed definitively as well.

## Diagnostic methods

Sequencing of the *GLI3 *coding exons or scanning with denaturing high performance liquid chromatography (DHPLC), single-strand conformation polymorphism (SSCP), or other conformation detection methods is an appropriate first screen for patients with typical GCPS. As noted above, the yield for mutations in patients with typical GCPS should be greater than 70%. For patients with subclinical forms of the disorder, it may be reasonable to use this same approach, although the yield is likely to be lower (Biesecker *et al*, unpublished data).

Cytogenetic analysis should be performed either as a first test, or in all patients who have GCPS but no mutation was found by sequencing. This is because translocations involving 7p have been shown to be the causative mutation in a small proportion of patients with GCPS. These mutations are important to detect because persons with balanced translocations have a risk for offspring with unbalanced translocations in addition to their risk of having a child with GCPS.

The broad range of insertions and deletions that can cause GCPS are technically challenging to detect. No current technology can be used to assess the full range of the deletions and insertions (duplications) that have been shown to cause this disorder. Sequencing and conformational analysis are useful for detection of aberrations that are significantly smaller than the polymerase chain reaction (PCR) amplicons used in these techniques. Fluorescence *in situ *hybridization (FISH), Southern blotting, loss of heterozygosity (LOH) analysis of polymorphic markers, quantitative PCR, and array comparative genomic hybridization (CGH) have all been used to detect duplications and deletions, with varying success (and varying practicality). FISH works well for deletions and duplications that are about 50% or more the size of the probes used in the assay. However, because the sizing of deletions is important for prognosis (see above) it can be tedious to determine this with FISH. We have developed an array CGH platform specifically designed to detect deletions and insertions in the *GLI3 *region, and this platform can readily assess aberrations of 10 kb and greater [16]. There is still a need for a mutation detection platform that bridges these technologies and it would, of course, be desirable to have a single platform that could detect all types of aberrations, though none are on the horizon.

## Differential diagnosis

The differential diagnosis for polydactyly is enormous, comprising more than 100 disorders and is beyond the scope of this chapter [17,18]. Therefore, the careful assessment of sometimes subtle dysmorphic features of a patient with polydactyly is key to making a correct diagnosis. The spectrum of GCPS overlaps with that of the so-called non-syndromic preaxial polydactylies such as preaxial polydactyly type 4 [19]. A few disorders have substantial overlap with GCPS. The acrocallosal syndrome comprises preaxial polysyndactyly, macrocephaly, agenesis of the corpus callosum, mental retardation, seizures, and hernias [20]. It is inherited in an autosomal recessive pattern; however, this fact is of little use in the differential diagnosis of a simplex case. There are two further complicating factors with acrocallosal syndrome. First, there is a single case of a patient with a phenotype indistinguishable from acrocallosal syndrome who has a p.A934P *GLI3 *mutation [21]. Second, patients with GCPS-CGS have substantial phenotypic overlap with acrocallosal syndrome [9,22]. In these situations, molecular diagnostics are essential to arrive at a correct diagnosis. The Teebi hypertelorism syndrome shares craniofacial manifestations with GCPS, although the polydactyly is typically not preaxial [23]. Carpenter syndrome manifests polysyndactyly and craniosynostosis, with mental retardation and has recently been shown to be caused by mutations in the *RAB23 *gene [24]. The Gorlin syndrome (nevoid basal cell carcinoma syndrome) also manifests macrocephaly, and occasionally manifests hypertelorism and polydactyly. Gorlin syndrome is caused by mutations in *PTCH1*, another gene in the GLI-SHH pathway [25].

## Recurrence risks

In multiplex families affected with GCPS, the recurrence risk for affected persons is 50%. The penetrance of GCPS is high, but is not 100% [7]. Therefore, unaffected persons from multiplex families have a risk for affected children that is probably less than 1% per conception. Apparently unaffected parents of simplex (apparently *de novo*) affecteds should be examined carefully for subtle signs of the disorder and molecular testing is indicated. If they manifest no signs of the disorder and do not carry the mutation seen in the affected child, they should be advised of a small recurrence risk, again probably less than 1% per conception. Gonadal mosaicism for GCPS has not been reported, but there is no reason to assume that this is not possible.

For multiplex families, the severity of the phenotype in that family is the best guide to the likely severity of future affected offspring in that family. That is because the intrafamilial variability is less than the interfamilial variability. It warrants re-emphasizing that the risk of cognitive impairment in GCPS caused by point mutations or deletions less than 1 Mb may not be significantly elevated over background rates. It is true that there are a modest excess of cognitively impaired children with GCPS among research cohorts, but again, ascertainment bias for increased enrollment of children with developmental disabilities may account for much or all of this elevation. Molecular diagnostics play a key role in the evaluation of simplex cases with manifestations compatible with GCPS-CGS or acrocallosal syndrome. Because the latter is inherited as an autosomal recessive trait, distinguishing these two diagnoses dramatically changes the recurrence risk (<1% *vs*. 25%) for the parents of the affected child. Although the gene for acrocallosal syndrome has not been determined, testing for a large deletion of the *GLI3 *locus can be critical to make this distinction.

As in all rare dominantly inherited disorders caused by loss of function mutations, the mutational spectrum of GCPS is broad and in some families it may be difficult to determine with certainty that the detected sequence variant is causative. These variants should be interpreted with caution.

## Antenatal diagnosis

Antenatal molecular diagnosis is technically straightforward to perform (*via *amniocentesis or chorionic villus biopsy) on at risk pregnancies in families where a causative point mutation has previously been determined. As noted in the previous section, some unique mutations detected in patients with GCPS can be difficult to interpret and in such cases, prenatal molecular diagnosis should be approached with caution. Antenatal diagnosis may also be undertaken for chromosomal aberrations that cause GCPS (translocations, large deletions, *etc*.) and the considerations for performing these tests antenatally are no different than those associated with other phenotypes.

Antenatal ultrasound may also be used to assess polydactyly. The ultrasound finding of macrocephaly is probably not sufficiently specific to be used for antenatal diagnosis of GCPS. High-resolution ultrasound can detect fetuses with polydactyly.

## Management including treatment

There are few associated medical complications of GCPS. Generally, the patients are healthy and have a normal lifespan. Surgical treatment of polysyndactyly should be considered, and in many cases leads to excellent outcome. As noted above, some patients with GCPS can have cognitive delays, although in GCPS these are typically mild. Proper developmental assessment and early intervention should be offered to any patient manifesting developmental delay. The nature of the intervention that is appropriate for patients with GCPS cannot be specified solely on the basis of the diagnosis. Instead, patients should be assessed individually and the appropriate interventions implemented.

## Prognosis

The prognosis of typical GCPS is good with a low or background rate of cognitive impairment, which is typically the most worrisome manifestation of the disorder among affected families. Polydactyly is straightforward to treat in many instances, although it can be complex when the patient has severe cutaneous syndactyly. Postaxial polydactyly is easier to repair than is preaxial polydactyly, and the latter should be performed only by experienced surgeons. Surgical repair of the hands is important for both functional and esthetic reasons. Our group is aware of a family where postaxial polydactyly type A was not repaired, as the digit was normally formed and fully functional. Surgical repair of the feet is optional in many cases as esthetic considerations are generally less pressing (compared to the hands) and the functional consequences of iatrogenic biomechanical problems can be severe.

## Unresolved questions

The complete spectrum of phenotypes attributable to *GLI3 *mutations has not been fully delineated, again because of ascertainment bias. This author suspects that there are a number of other candidate phenotypes, such as oral-facial-digital syndromes, that may be caused by *GLI3 *mutations. As well, *GLI3 *mutations may cause non-syndromic polydactyly, although this diagnosis can be difficult to make with confidence [19]. As noted above, not all patients with GCPS have *GLI3 *mutations and molecular diagnostic tools to assess these patients would be clinically useful.
